# Confidence in managing open-globe injuries and endophthalmitis: a pre- and postwet-lab training evaluation

**DOI:** 10.3389/fopht.2026.1740823

**Published:** 2026-06-17

**Authors:** Vegard Asgeir Forsaa, Birger Lindtjørn

**Affiliations:** 1 Department of Ophthalmology, Stavanger University Hospital, Stavanger, Norway; 2 Department of Clinical Medicine, Section of Ophthalmology, University of Bergen, Bergen, Norway; 3 Department of Quality and Health Technology, University of Stavanger, Stavanger, Norway

**Keywords:** active learning, corneal penetration, endophthalmitis, ocular trauma, openglobe injury, penetrating eye trauma, porcine lab, wet lab

## Abstract

**Purpose:**

The purpose of this study was to evaluate the effect of 2 days of wet-lab training on confidence in the management of open-globe injury (OGI) and endophthalmitis.

**Methods:**

In a porcine wet lab, 24 participants were trained on 12 surgical tasks, including pars plana vitreous sampling and corneal and scleral suturing. Participants anonymously rated their confidence in managing OGI and endophthalmitis on a scale from 1 to 10 before the course, immediately after its completion, and 6 months later.

**Results:**

Confidence in corneal suturing improved from a median of 1 (interquartile range [IQR] = 1) to 7 (IQR = 3) immediately after the course (*p* < 0.001), and to 3 (IQR = 2) six months later (*p* = 0.001). For scleral suturing, confidence improved from a median of 1 (IQR = 1) to 7 (IQR = 2) (*p* < 0.001), and to 3 (IQR = 3) 6 months later (*p* = 0.001). Confidence in endophthalmitis treatment improved from a median of 5 (IQR = 5) to 8 (IQR = 4) immediately after the course (*p* < 0.001), and remained at 8 (IQR = 2) 6 months later (*p* = 0.012).

**Conclusions:**

Wet-lab training effectively increased participants’ confidence in managing OGI. Key elements of the ophthalmic surgeon’s learning curve can be addressed in a porcine wet lab, thereby reducing the risk of poor patient outcomes. Six months later, the positive effect on confidence remained significant, despite the expected decline over time. Nonetheless, there remains scope for further improvement in these essential surgical skills.

## Introduction

Open-globe injuries (OGI) and endophthalmitis are ocular emergencies that often result in severe visual loss. Corneal and scleral wounds expose vulnerable intraocular tissues to environmental bacterial and fungal flora. Prognosis critically depends on closing the globe within 12 to 24 h, as delayed closure increases the risk of endophthalmitis (1–3).

OGI and endophthalmitis pose a challenge for all ophthalmologists on on-call duty. In Norway, many small eye departments in rural areas face this challenge particularly acutely. Referral to a larger eye department introduces time-consuming logistics and long-distance travel, which compromise prognosis. Optimal care requires closing the globe and treating endophthalmitis at the local eye department as soon as possible. This underlines the importance of relevant surgical skills for all ophthalmologists on duty. Fortunately, strict regulations on health, safety, and environment, together with improved road and traffic safety in industrialized countries, have contributed to a reduction in OGI (4). Nonetheless, this favorable development also comes at a cost. Ophthalmologists struggle to acquire the surgical skills necessary for managing OGI. In a UK survey, 43% of ophthalmology residents had not treated a corneal laceration, and 60% had never repaired an OGI (5). In the USA, the situation appears somewhat better, but only 65% of residents reported confidence in managing OGIs (6). Furthermore, under the traditional apprenticeship model of learning, patients have borne the burden associated with the surgeon’s learning curve. Today, society has little tolerance for unnecessary complications arising from these circumstances (7, 8). To overcome these challenges, effective methods are needed to improve the essential surgical skills of ophthalmology residents in a safe and efficient manner.

Workshops are widely used to improve confidence and performance in procedural skills among medical students (9, 10). In wet labs, the use of fresh animal tissues enables the simulation of various cases in a realistic environment. This risk-free setting provides extensive exposure to surgical procedures and has been proven to enhance surgical capabilities (11, 12). In ophthalmic surgery, wet labs have been demonstrated to strengthen microsurgical skills across a range of procedures (13, 14).

Established in 2016, a porcine wet-lab course became mandatory for ophthalmology residents in Norway by 2024. Participants practice various scenarios of OGI and endophthalmitis in a realistic surgical setting. This study aimed to investigate the impact of 2 days of wet-lab training on confidence and motivation in acquiring the surgical skills necessary for OGI and endophthalmitis treatment.

## Materials and methods

**Figure 1:**
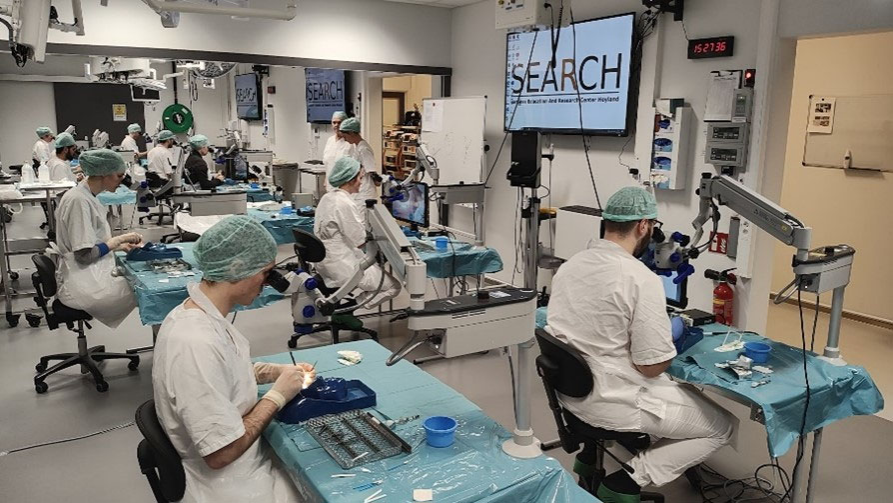
Wet-lab setting.

**Figure 2:**
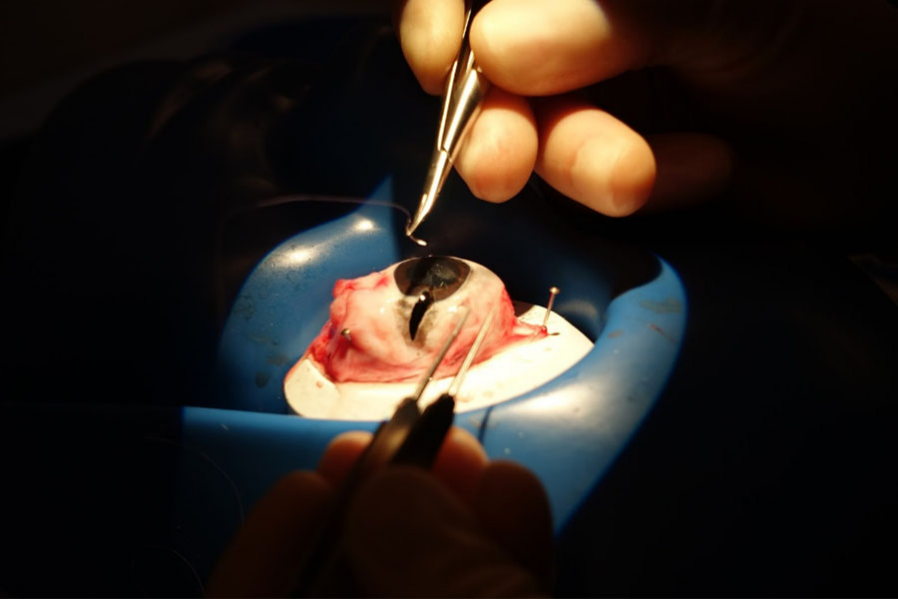
Open-globe injury with corneal penetration crossing the limbus.

The admission criteria for the wet lab constituted the study’s inclusion criteria: final−year ophthalmology residents who had not previously taken the course. Priority was given to those with the least time remaining in their training. Although written consent was not obtained, participation was entirely voluntary, and all participants received written information about the study. The wet lab accommodates 12 participants and is located at Sandnes Research Center Høyland at the Norwegian University of Life Sciences in Sandnes, Norway. Twelve stations are equipped with surgical microscopes (Carl Zeiss Opmi Pico; Carl Zeiss Meditec AG, Jena, Germany) and the following surgical tools: needle drivers; three types of scissors (Vannas, iris, intraocular); four types of forceps (tying, surgical, intraocular internal limiting membrane, and intraocular serrated); a 1.2-mm side port lance; viscoelastics; trocars; vitrectomy probes (not connected to a machine); syringes and tips (sharp and blunt), and sutures with curved, straight, and long curved needles (10–0 Ethilon, 7–0 Vicryl, 10–0 Prolene; Ethicon, LLC, Puerto Rico, USA; 10-0 polypropylene; Mani Inc., Tochigi, Japan). Freshly exenterated porcine eyes were placed in a facial-shaped model (Head 4 Eyes; Eyecre GmbH, Kematen, Austria) (**Figures 1**, **2**). The porcine eyes were obtained from slaughter pigs at a nearby slaughterhouse.

### Passive learning sessions

Five lectures of 30 min each covered the following topics: suturing techniques, classification and theory of ocular trauma, anterior segment trauma, posterior segment trauma, and endophthalmitis.

### Active learning sessions

Over the 2 days, participants received 12 h of wet-lab training. They were provided with a list of 12 surgical tasks distributed across the four topics: corneal suturing, scleral suturing, managing endophthalmitis, and iris suturing (**Table 1**). Two instructors were always available, and all procedures were completed entirely by the participants.

**Table 1:** Surgical tasks.

Corneal and scleral penetrations	Endophthalmitis	Iris defects
Straight corneal 4 mm	Placing a pars plana trocar	Sector defect with McCannel’s suture
Straight corneal 8–10 mm	Vitreous fluid sampling by vitrector	Sector defect with Siepser’s sliding knot
Limbal corneoscleral crossing	Injection of antibiotics	Dialysis with mattress sutures
Oblique corneoscleral limbal crossing		Atonic pupil with iris cerclage
Complex corneal V and Y shapes		
Complex corneal X, W, and Z shapes		
Lamellar corneal		

### Questionnaire

To our knowledge, validated questionnaires in the pedagogy of surgery are lacking. Therefore, a Likert-type questionnaire was developed specifically for this study by the first author, focusing on participants’ confidence and motivation regarding four surgical tasks, and on confidence alone for three general microsurgical skills (**Table 2**). Likert scales are widely used to measure subjective constructs such as attitudes and confidence (15, 16). The validity and reliability of this questionnaire have not been assessed; however, internal consistency for the confidence and motivation constructs was calculated. Participants anonymously rated their confidence and motivation on a scale from 1 to 10 before the course and immediately after its completion. A final questionnaire was administered as an anonymous electronic poll 6 months later. Participants were also asked to report which surgical tasks they had performed during the 6 months following the course and whether they had served on ophthalmological on-call duties.

**Table 2:** Questionnaire.

	Tasks				Skills		
	Corneal sutures	Scleral sutures	Endophthalmitis treatment	Iris sutures	Microscope surgery	Eye–hand coordination	Surgical knots
Confidence	To what extent do you feel capable of suturing corneal penetrations?	To what extent do you feel capable of suturing scleral penetrations?	To what extent do you feel capable of treating acute endophthalmitis?	To what extent do you feel capable of suturing iris defects?	To what extent do you feel capable of performing surgery through a microscope?	To what extent do you feel you master eye–hand coordination?	To what extent do you feel you master surgical knots?
Motivation	How motivated are you to suture corneal penetrations when you have on-call duty?	How motivated are you to suture scleral penetrations when you have on-call duty?	How motivated are you to treat acute endophthalmitis when you have on-call duty?	How motivated are you to suture iris defects?			

### Statistical analysis

Data were summarized as median and interquartile range (IQR; 25th to 75th percentiles). The Wilcoxon signed-rank test was used to compare pre- and postcourse ratings. A two-sided *p*-value ≤ 0.05 was considered statistically significant. Internal consistency reliability of the questionnaire was assessed with Cronbach’s alpha. All statistical analyses were performed using SPSS software, version 26 (IBM Corp., Armonk, NY, USA). Boxplots were generated in GraphPad Prism, version 10 (GraphPad Software, San Diego, CA, USA).

## Results

Twenty-four participants—12 from the October 2024 course and 12 from the February 2025 course—contributed to this study. The cohort comprised 15 female and nine male participants, all of whom were final−year ophthalmology residents.

**Figure 3:**
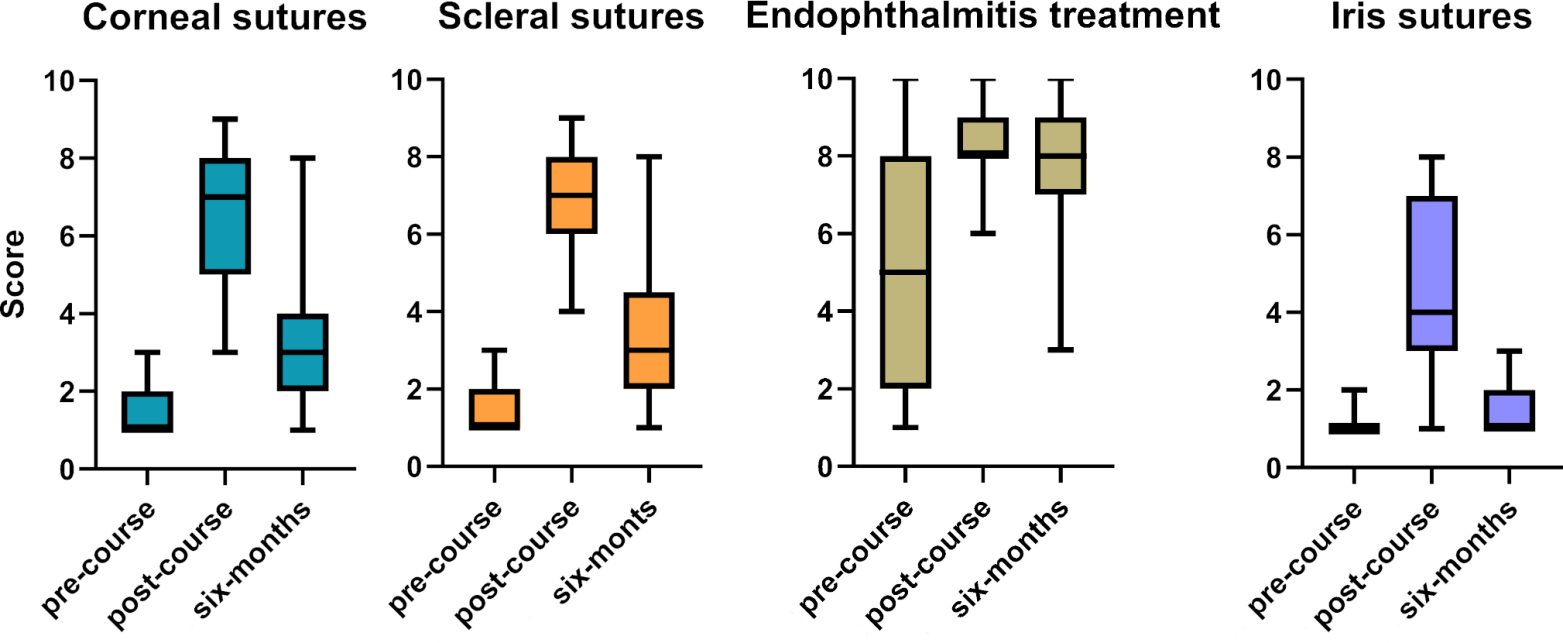
Boxplot of participants’ rated confidence in specific surgical tasks. Each box represents the interquartile range (IQR), the vertical line inside the box indicates the median, and whiskers extend to the lowest and highest values.

**Figure 4:**
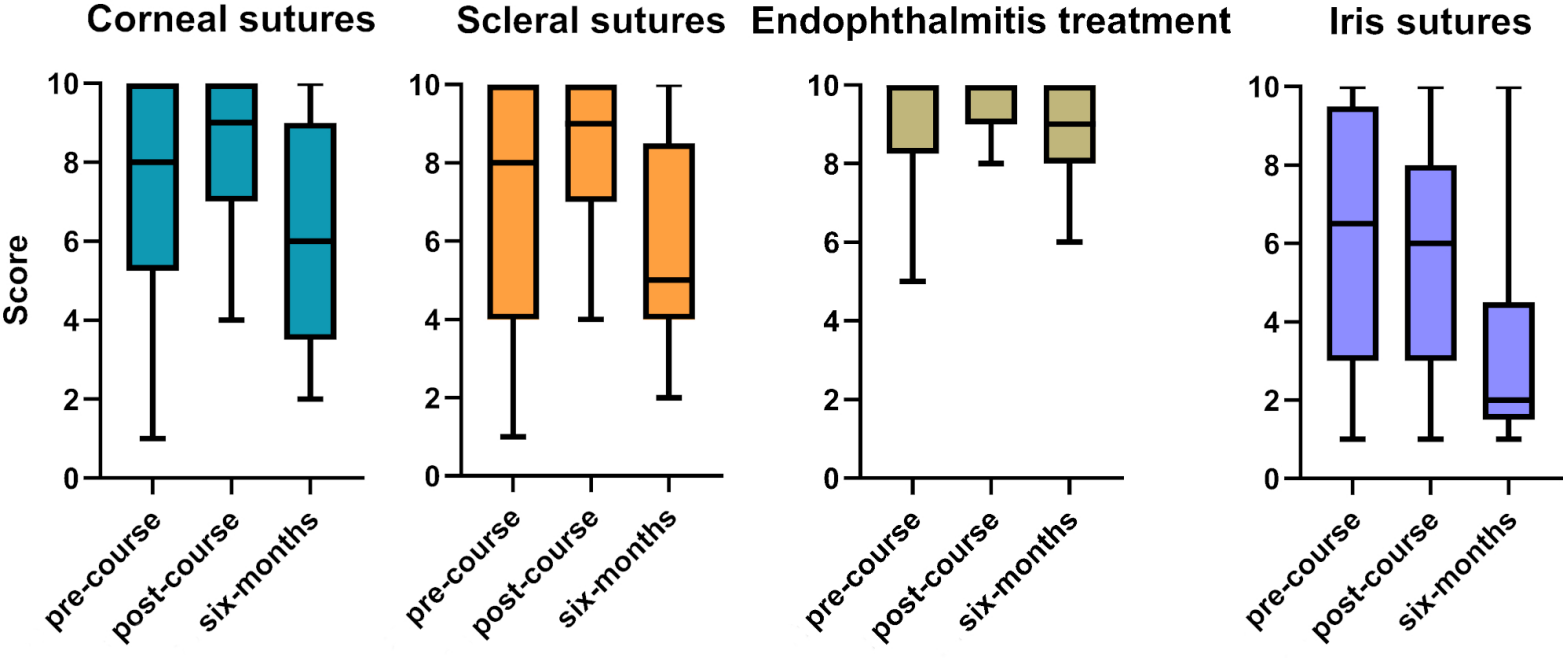
Boxplot of participants’ rated motivation in specific surgical tasks. Each box represents the interquartile range (IQR), the vertical line inside the box indicates the median, and whiskers extend to the lowest and highest values.

**Figure 5:**
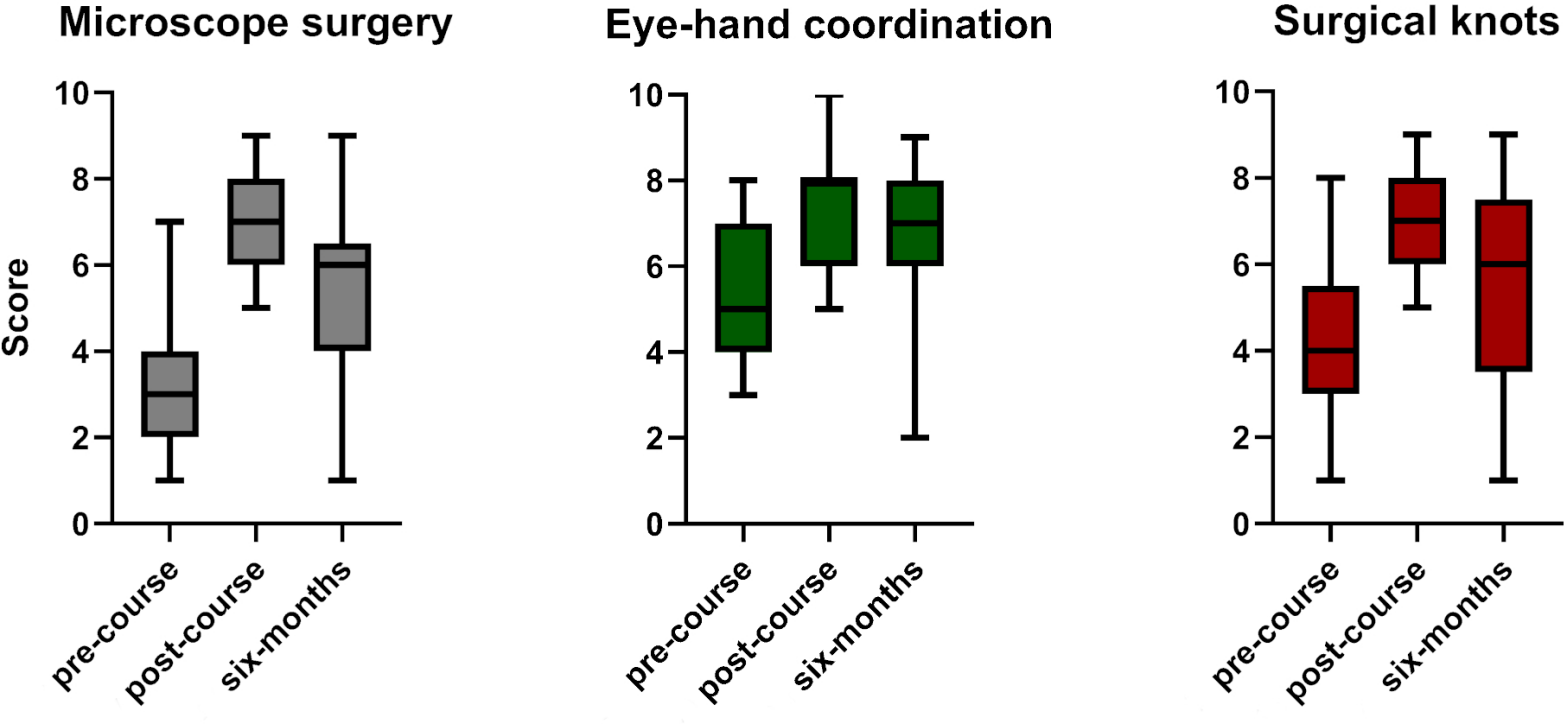
Boxplot of participants’ rated confidence in general surgical skills. Each box represents the interquartile range (IQR), the vertical line inside the box indicates the median, and whiskers extend to the lowest and highest values.

The results of the anonymous questionnaires are presented in **Tables 3**, **4**, **Figures 3**–**5**. Confidence levels showed a highly significant increase across all four tasks and the three general skills from course initiation to completion. At 6 months, confidence had declined but remained significantly higher than at course start for all tasks (corneal sutures, *p* = 0.001; scleral sutures, *p* = 0.001; endophthalmitis treatment, *p* = 0.012; iris sutures, *p* = 0.014). For the general skills, only microscope surgery retained significantly higher confidence after 6 months (*p* = 0.004), whereas eye–hand coordination (*p* = 0.107) and surgical knots (*p* = 0.095) did not. Notably, precourse confidence in endophthalmitis management was considerably higher than for the other three tasks and remained the highest rated after 6 months. Conversely, confidence in iris suturing was considerably lower than for the other tasks, both at course completion and at 6 months.

**Table 3:** Rating of confidence.

	Tasks				Skills		
	Corneal sutures	Scleral sutures	Endophthalmitis treatment	Iris sutures	Microscope surgery	Eye–hand coordination	Surgical knots
Start (24/24)	1 (1)	1 (1)	5 (5)	1 (0)	3 (2)	5 (3)	4 (2)
Completion (*p*-value vs. start, 24/24)	7 (3) **< 0.001**	7 (2) **< 0.001**	8 (4) **< 0.001**	4 (4) **< 0.001**	7 (2) **< 0.001**	8 (2) **< 0.001**	7 (2) **< 0.001**
After 6 months (*p*-value vs. start, 17/24)	3 (2) **0.001**	3 (3) **0.001**	8 (2) **0.012**	1 (1) **0.014**	6 (3) **0.004**	7 (2) 0.107	6 (4) 0.095

The results are expressed as median ratings with IQR. Questionnaire ratings were based on a scale from 1 to 10. Values in bold indicate statistical significance.

**Table 4:** Motivation ratings.

	Tasks			
	Corneal sutures	Scleral sutures	Endophthalmitis treatment	Iris sutures
Start (24/24)	8 (5)	8 (6)	10 (5)	6 (6)
Completion (*p*-value vs. start, 24/24)	9 (3) 0.144	9 (3) 0.085	10 (1) 0.183	6 (9) 0.750
After 6 months (*p*-value vs. start, 17/24)	6 (6) 0.361	5 (5) 0.154	9 (2) 0.567	2 (3) **0.013**

The results are expressed as median ratings with IQR. Questionnaire ratings were based on a scale from 1 to 10. Values in bold indicate statistical significance.

There were no significant differences in motivation ratings across the four task categories from course start to completion (**Table 4**). At 6 months, however, the motivation self-rating had decreased significantly for the iris suturing task. Endophthalmitis treatment achieved the highest motivation rating at 6 months.

Of the 17 participants who responded to the questionnaire, 13 were serving ophthalmological on-call duties at 6 months. Eight of these 13 participants reported applying the rehearsed surgical tasks within the 6 months following the course. In total, they had treated six cases of endophthalmitis, two corneal penetrations, and one scleral rupture during this period.

The internal consistency reliability for confidence was 0.642, 0.842, and 0.831 at course start, course completion, and 6 months later, respectively. For motivation, the corresponding values were 0.888, 0.843, and 0.735.

## Discussion

The main finding in this study was a clear increase in confidence ratings for surgical task execution during the course (**Tables 3**, **4**; **Figures 3**, **5**). After 6 months, confidence across all surgical tasks remained significantly higher than at course start, although it was considerably lower than immediately after the course. Motivation scores were high at the beginning of the course and remained so throughout its duration; however, as anticipated, they had generally declined by the 6-month follow-up (**Table 4**; **Figure 4**).

When focusing more specifically on the individual surgical tasks, endophthalmitis treatment showed an extremely high motivation score at the beginning, which remained very high over 6 months (**Table 3**; **Figure 3**). This was accompanied by a marked rise in confidence scores from an already moderately high baseline, which also remained very high over 6 months. Notably, among the scenarios that were rehearsed, endophthalmitis was the most frequently treated condition in the 6 months postcourse, a factor that may have positively influenced participants’ motivation. The motivational impact on learning is closely related to the perceived value of the educational task. When this value is perceived as high and aligned with the learner’s goals, participants are more likely to focus and engage in deep learning (17).

The corneal and scleral suture tasks showed a huge rise in confidence scores, starting from a very low baseline at the beginning of the course (**Table 3**; **Figure 3**). Thereafter, a decline in confidence was observed at 6 months, although scores remained significantly higher than the precourse score. The significant increase in confidence score reflects a steep learning curve arising from trial-and-error learning under supervision. Trial-and-error learning improves memory and provides mnemonic advantages from errors made (18). These errors represent important preconditions for effective learning, as they allow for self-reflection. In a stepwise manner, surgical skills are constructed on newly acquired expertise. Although significantly improved compared with baseline, the 6-month confidence rating of only three out of 10 for corneal and scleral suturing clearly highlights the need for further skill development. We also observed that the motivation score for the corneal and scleral suture task was not as high as that of the endophthalmitis treatment task after 6 months (**Table 4**; **Figure 4**). This may be attributed to greater complexity and the lower frequency of corneal and scleral suture tasks.

The iris suture task stands out with the lowest ratings for both confidence and motivation over the whole study period. Notably, iris suturing is less relevant in the acute phase of trauma cases and is often managed by anterior segment surgeons at a later stage. We do not consider iris suturing to be a core skill, and therefore place less emphasis on it in the course. Nevertheless, this task offers additional surgical experience in the anterior segment of the eye, which broadens the surgical capability and expertise in tissue handling.

In this wet lab, 83% of the time was devoted to solving 12 different surgical tasks, representing active learning (**Table 1**). The remaining 17% consisted of traditional lectures, typical of passive learning. Active learning is well documented as a superior method of acquiring knowledge, and porcine wet labs in particular have demonstrated verified advantages for the development of surgical skills across various environments (19). Surgical residents training in thoracic surgery, trabeculectomy, and laparoscopic skills in urology are just some examples (20–22). In a wet lab like this, the surgical scenarios can be very realistic, and the setting is very comfortable, with no risk of inflicting harm on patients. This creates a favorable environment for learning. In the opposite situation, negative feelings and stress have been shown to negatively impact the learning process (23).

Among the limitations of this study are the low number of participants and the fact that only 17 of 24 completed the 6-month questionnaire. Responders may have been more supportive than nonresponders, introducing a bias towards higher ratings of motivation and confidence. The questionnaires were nonvalidated, and although Cronbach’s alpha indicated high internal consistency for confidence at course completion and 6 months later, this was not observed at course start. This introduces uncertainty into the interpretation of the confidence rating results, particularly for baseline confidence. Also, different levels of surgical experience at baseline may have contributed to bias and were not controlled for. In addition, the self-reported confidence levels do not equate to objective assessments of surgical competence; therefore, the actual surgical expertise of the participants remains uncertain. Further research employing objective assessments of technical skill acquisition is warranted, including metrics such as time to complete a standardized suturing task, blinded expert ratings of sutured wounds, or evaluation using a validated OSATS scoring system (24).

Based on the self-ratings, the wet lab significantly enhanced participants’ confidence in managing surgical tasks related to OGI and endophthalmitis during the 6 months following the course. Elements of the ophthalmic surgeon’s learning curve could effectively take place in a porcine wet-lab setting, thereby reducing the likelihood of suboptimal patient outcomes. However, there remains clear potential for further improvement in the critical surgical skills.

## Data Availability

The raw data supporting the conclusions of this article will be made available by the authors, without undue reservation.
